# Understanding Global Health: An Experience from International Professional Exchange Program

**DOI:** 10.31729/jnma.5380

**Published:** 2020-09-30

**Authors:** Bibechan Thapa

**Affiliations:** 1 KIST Medical College and Teaching Hospital, Mahalaxmi, Lalitpur, Nepal

Medical education is becoming highly advanced and equally challenging. To excel here, a strong grasp of theoretical knowledge,as well as itsproper implementation in medical practice is a must. During the internship period, medical graduates are exposed to complete clinical and practical aspects of medical science. This is crucial, as the knowledge gained in medical school is finally put totest during patient care. Acknowledging this, medical schools in Nepal provide a one-year compulsory rotational internship, where recent graduates receive ample learning opportunities through first-hand exposure to cases in the various departments of a hospital.

The MBBS curriculum of Nepal focuses on producing medical professionals with sound knowledge and skills, however changing time demands changing approaches. In today's world, integrating global health in medical schools has been of utmost importance. Many medical schools in countries like the United States and Canada have brought changes to their curricula to address this situation. The medical institutions of Nepal should give a serious thought on rectifying this much-needed issue.

## WHAT IS GLOBAL HEALTH?

The term “Global health” is inclusive of all health issues that surpass class, color, culture, creed, economy, and borders and thus is a global common ground of health. It has evolved out of the concept that although disease patterns vary geographically, factors like namely poverty, illiteracy, limited access to health care, gender disparity, environmental changes, political instability, war, and genetic susceptibility influences the spread and onset of diseases.^1^ Also, global environmental changes, international trade, movement of people, pathogens, and other social and economic forces play a fundamental role in health and access to health care.^2^ A most recent example of a global health issue is the COVID-19 pandemic which has taught us a good lesson and made us critically think about global health.

Globalization has transformed the determinants of health as well as health care delivery quite radically and irreversibly. This changing dynamic has ignited students to be acquainted with global health issues, which despite the growing interest, medical education programs of many countries have been unable to provide.^2^ Medical education of Nepal is far from addressing this issue of importance as it is still following all conventional syllabus that too was designed decades ago and hasn't been upgraded promptly.

By bringing together interested individuals and providing the necessary resources and platforms, it is possible to develop programs that provide exciting global health education opportunities for medical students and professionals. One exemplary form of the same is an international student and professional exchange programs,the interest of which has been consistently increasing among medical students.^2^

Medical schools of leading countrieslike the USA and Canada are re-evaluating their global health curricula andare endorsing international exchange programs as a key part of the global health movement, as they understand the educational needs and benefits of participation in international electives are significant.^1^ Medical schools of Nepal have to adopt such a policy if they wish to produce doctors who are well trained and versatile enough to adapt to ever-changing international medical standards and practices.

## MERITS OF EXCHANGE PROGRAMS

Many studies have established the merits and have made the recommendations of such programs. At the end of the program, a majority of participants noted that the exposure not only improved their clinical diagnostic and communication skills, but also changed world views, increased cultural sensitivity, enhanced community, social, and public health awareness, appropriate resource utilization; shifted career choices, reduced their dependence on laboratory and other procedures and brought a greater understanding of the opportunities and challenges of working in areas with resources different from theirs. On self-assessments, up to 83% of the students said this experience changed how they practiced medicine and almost 96% of the students recommended international health electives to other students.^3^ Bearing in mind its varied merits international exchanges must be promoted in Nepal as well.

## IMPORTANCE

The students' reflections on this experience indicated that studying an alternative medical. The students' reflected that studying the non-native medical system, in its local setting, prepared them to open to other medical practices, beliefs, and cultures in healthcare delivery. Also, facing a linguistic barrier themselves helped the students empathize with patients of limited language proficiency.^4^ Thus, such diverse exchanges shape medical students into more clinically and culturally competent professionals.

There has been a significant disparity in medical students' knowledge about healthcare systems across countries, and to address this, countries like the United States, Germany, etc developed an innovative solution. Hence, the United States-European Union Medical Education Exchanges (US-EU MEE) program was launched. The primary focus of this exchange program is to assimilate international health care policies by having the student learn from the practices of the host country. This program has been an effective means of introducing global health issues in their curriculum.^5^ Countries like the US and those of the EU, despite having highly advanced medical systems, believe that they have deficits in their health care system and the exchange programs will help highlight the issues while benefiting the participants. The health care system of developed countries and developing countries like ours have huge disparities. Thus exchange programs between these countries will play a significant role in understanding and addressing a global health issue.

Students who have participated in the exchange program have reported uniformly that the experience was rewarding, educational, and instructive. With its patient-based learning, there are opportunities to observe first-hand socio-medical and socioeconomic issues that a typical patient presents. It has helped students gain better insight regarding service delivery, financing, strengths, and weaknesses of another health care system, which is a learning opportunity not only to the individual but also to the institution as a whole.^5^

Global outbreaks like Ebola, SARS, MERS, and COVID-19 have highlighted the importance of coordinated global health response.^6^ Exposure to international health care systems ensures that we, as to-be professionals, understand the characteristics and complexities of the global health system. Besides global outbreaks gaining first-hand exposure to diseases very rarely encountered in a part of the world and learning about technological advancements is also its major benefits. The epidemiology of diseases, disparities in global health systems, and the importance of cross-cultural sensitivity must not be neglected; for which global health training and international clinical rotations are of utmost importance.^7^ Professional and research exchanges of a massive scale are being provided by the many universities in the US and Europe, and it is increasingly being adopted in many Asian countries as well.

## OPPORTUNITIES

International Federation of Medical Students Associations (IFMSA) runs the Professional Exchange program which is a full educational program offering clerkships to medical students abroad, also is endorsed by the World Federation of Medical Education (WFME), and many others. Each year, more than 15,000 medical students embark on this journey.^8^ These exchange programs are key promoters of intercultural understanding and cooperation amongst medical students and health professionals, which is of much significance in today's globalized world. Thus, such a highly recognized program is a golden learning opportunity for exploring and experiencing medical education and practices in a completely different setting.

Medicine is a field where things, even facts are constantly changing and evolving. From trivial alterations in information to new innovative methods of diagnosis and treatments, research has played a major role in such undertakings. But, many medical students, especially from a low-resource setting like ours, do not have the opportunity to foster research. IFMSA also has a research exchange program similar to the professional exchange program.^8^ These sorts of opportunitieshave helped develop culturally sensitive students and skilled researchers.

Nepal Medical Student Society (NMSS) is working with IFMSA and providing opportunities to Nepalese medical students to be part of such a program. This program has fostered cooperation and collaboration among medical students by breaking down social barriers, promoting opportunities for dialogue, and creating clinical exchanges.

I had a chance of getting enrolled in the professional exchange program, definitely a life experience with steep learning curve. I was enrolled in a month-long clinical clerkship program at the Department of Anesthesia and Intensive Care Medicine, Third Faculty of Medicine, Charles University, and Fakultni Nemocnice Kralovske Vinohardy University hospital in Prague, Czech Republic. The Czech Republicis a landlocked country like ours located in Central Europe. Health status in the Czech Republic has improved significantly over recent years with marked improvements in life expectancy and mortality, both closer to the European average.^9^ The Health system of Czech was struggling a decade ago however they commendably rectified it. By critically analyzing and adapting their policies Nepal can transform its health care system.

During this clerkship, I also met delegates from countries like Germany, Cambodia, the United Kingdom, and Columbia. Sharing experiences with them further made me appreciate the importance of this program. While it seems an alien concept of Nepalese medical students,it forms an integral part of their curriculum and is highly encouraged upon. For many of them, it wasn't their first time, and naturally, they were quite acquainted with the ways of interacting and learning optimally from the program. Getting to know about the health system of other countries through directly observing and being part of it, helps to figure out the flaws in our health system and make the necessary recommendation for improvements.

## CHALLENGES

Some students go for a professional exchange from Nepal, but they are unfortunately unable to get the equivalence of the rotation from the university. Exchanges help make students change-makers and enable them to make an impact in global health with the knowledge gained and inter-personal connections formed in the course of their exchange. Thus, it has the potential to play a significant role in the changes we need to make in the health care system of Nepal.^10^ The program's strengths are multitudinous; however, the lack of a formal evaluation is one of the very few weaknesses that will creep up while establishing this system of learning. We need to be vigilant towards this to ensure an optimal outcome.

## WAY FORWARD

All students should get the opportunity to learn about global health, and gain first-hand experience by participating in professional and student exchange program. Medical schools should encourage while universities should work towards providing equivalence: a major hurdle for any medical students moving in this path.

## Conflict of Interest

**None.**
